# Public awareness, patterns of use and attitudes toward natural health products in Kuwait: a cross-sectional survey

**DOI:** 10.1186/1472-6882-14-105

**Published:** 2014-03-19

**Authors:** Abdelmoneim Awad, Dana Al-Shaye

**Affiliations:** 1 Department of Pharmacy Practice, Faculty of Pharmacy, Kuwait University, Kuwait City, Kuwait; 2 Ministry of Health, Kuwait City, Kuwait

**Keywords:** Natural health products, Herbal remedies, Patterns of use, Kuwait

## Abstract

**Background:**

There has been a global rise in the use of natural health products (NHPs). Proper regulation of NHPs is pivotal to ensure good quality control standards, enhance consumers' safety and facilitate their integration into modern healthcare systems. There is scarcity of published data on the prevalence of NHPs usage among the general Kuwaiti population. Hence, this study was designed to determine awareness, patterns of use, general attitude and information requirements about NHPs among the public in Kuwait.

**Methods:**

A descriptive cross-sectional survey was performed using a pretested self-administered questionnaire on a sample of 1300 Kuwaiti individuals, selected from six governorates in Kuwait using a multistage stratified clustered sampling. Descriptive and multivariate logistic regression analysis were used in data analysis.

**Results:**

The response rate was 90.2%. NHPs were thought to be herbal remedies by most of participants (63.5%), followed by vitamins/minerals (40.5%), traditional medicines (21.1%), probiotics (14.9%), amino acids and essential fatty acids (7.2%), and homeopathic medicines (5.6%). NHPs usage was reported by 71.4% (95% CI: 68.8-74.0%) of respondents, and mostly associated with females (OR: 1.90; 95% CI: 1.44-2.51). Herbal remedies were the most commonly used (41.3%; 95% CI: 38.5-44.2%). The most common reasons for using NHPs were to promote and maintain health and to prevent illness and build immune system. Family members and/or friends and mass media were the main sources for providing information about NHPs. About 18% of consumers have experienced a side effect due to using a NHP. Attitudes toward NHPs were generally positive; with more than 75% of participants believing that the Ministry of Health in Kuwait should regulate the claims made by the manufacturers of NHPs and it is important to talk to a medical doctor or a pharmacist prior to using NHPs. Most of the respondents showed increased interest to acquire knowledge about different types of information related to NHPs.

**Conclusions:**

The prevalence of use of NHPs among Kuwaiti population is high. The present findings have major public health policy implications for Kuwait. Therefore, there is an apparent need to establish effective health education programs and implement better and more regulated NHPs use policies in Kuwait.

## Background

Natural health products (NHPs) are defined as “*vitamins and minerals, herbal remedies, homeopathic medicines, traditional medicines such as traditional Chinese medicines, probiotics, and other products like amino acids and essential fatty acids*” [1]. They are made from ingredients found in nature that are promoted for use to prevent, diagnose, or treat disease; restore or correct function; or maintain or promote health [2]. There has been a global rise in the use of NHPs, several studies worldwide have examined the prevalent use of complementary and alternative medicine (CAM) including NHPs. Some of these studies have focused in patients with chronic diseases such as inflammatory bowel disease, cancer, hypertension and diabetes mellitus [3-14], while others have studied general populations [15-26].

In developed countries, a relatively high percent of NHPs usage have been reported. For instance, more than 70% of Canadians reported having used a NHP at some time in their lives [15,16]. While the reported rates of daily or weekly use of herbal and vitamin/mineral supplements in the United States were 55.4% and 43.1% in older women and men, respectively [17]. The prevalence of CAM use among U.S. adults and children was 40.0% and 11.8%, respectively [18]. A study from Australia showed that prevalence of CAM use in women was 90.5%. The two most commonly reported CAM used are vitamins/minerals (80.9%) and herbal medicines (40.9%) [19]. In south Australia, CAM therapies were used by 52.2% of the population, with vitamins (39.2%) and herbal medicines (20.6%) as the most used therapies [20]. The prevalence of CAM usage among children and adolescents in Wales was found to be 38.0%, with vitamins/minerals and herbal therapies being the most commonly used [21]. The analysis of studies conducted in Europe during the last 10 years showed that over 100 million of the citizens are CAM users [22].

The Eastern Mediterranean Region hosts one of the fastest growing markets of NHPs in the world [27]. Previous studies focused on patients with various forms of chronic illnesses showed prevalence of use varies from 7.6% to 85.7% in Palestine, Saudi Arabia, Oman, Morocco and Jordan [5,6,8-14]. Only few reports have indicated extensive use (43.2%-76.0%) of herbs among the general population in the United Arab Emirates and Saudi Arabia [23-26].

Several factors are associated with the growing popularity of NHPs, including individual dissatisfactions with conventional treatments, desire for greater autonomy and empowerment about healthcare choices, chronic diseases and cultural influences [28,29]. Other reasons include improving health and well-being, relieving symptoms associated with chronic diseases or side effects of conventional drugs, and preventing illness [9,16,18,20]. Moreover, NHPs use is increasing as a result of a widespread perception among the users that the preparations are natural and, therefore, safe and not to be associated with risk [30,31]. Several reviews had documented that NHPs, especially herbal products, are associated with adverse drug reactions [32-34]. The increased prevalence for the concomitant use of herbal and conventional medicines is an area of great concern due to its potential for medicine-herb interactions [23,35,36]. Furthermore, various incidents of adulteration of herbal remedies with convention medications, poor product quality, side effects and drug interactions were also reported from the Gulf region [37,38]. The increasing widespread use of traditional medicine (TM) has prompted the World Health Organization (WHO) to promote the global integration of traditional and complementary medicine into the national health care systems and to encourage the development of national policies and regulations [28]. Proper regulation of NHPs is pivotal to ensure good quality control standards, promote their safe and effective use, and facilitate their integration into modern healthcare systems. Also health care professionals should be in a better position to help patients make informed decisions about using these products and to minimize the risk of adverse reactions [28,34].

The state of Kuwait started regulation of herbal medicines in 1989. They are regulated as over the counter medicines, dietary supplements, health foods and functional foods. Regulatory requirements for the manufacture and safety assessment of herbal medicines are similar as for conventional medicines [39]. In Kuwait, the Ministry of Health established official guidelines for registration of NHPs including herbal medicines, vitamins/minerals, aminoacids and essential fatty acids, and homeopathic medicines under Herbal and Natural Medicine registration decrees [40]. The exact prevalence rate of NHPs usage among the general population in Kuwait is not known since there are limited published studies. One study reported CAM therapies usage among medical and pharmacy students, which was found to be 55.2% with herbal products being the most commonly used (37.6%). Massage, herbal products and prayer/Qur’an reciting were perceived by the students as the most effective, while cauterization as the most harmful. The students’ attitude toward CAM was positive; with 79.7% believing that CAM includes ideas and methods from which conventional medicine could benefit [41]. In view of the recognition that NHPs in its various forms is enjoying a growing popularity among the public in the Gulf region, coupled with the fact that there is a lack of information on the prevalence, socio-cultural and personal factors (knowledge/beliefs, familiarity, attitudes) underlying an individual’s decision to use a NHP. We hereby report a study designed to determine awareness, patterns of use, general attitude and information requirements about NHPs among the public in Kuwait.

## Methods

A descriptive, cross-sectional survey was conducted in Kuwait, a Middle-Eastern country with an area of 17,820 km^2^ and an estimated population of 3,328,136 people, one-third of which are Kuwaitis (2008 estimate). The survey was conducted during the period from October 2010 to August 2011. The study population consisted of Kuwaiti individuals from six governorates of Kuwait, namely, Al-Ahmadi Al-Farwaniyah, Al-Jahra, Capital, Hawalli and Mubarak Al-Kabeer. The ethical clearance for this study was obtained from the “Human Ethical Committee, Health Sciences Center, Kuwait University”.

The sample size was determined using PS power and sample size calculator V.3.05 [42]. A total sample size of 1080 individuals would be required to determine a 10% difference in proportion between two groups, for example, male versus female with 90% power and at 5% significance level. Assuming a response rate of 80%, a sample size of 1300 was selected using multistage stratified clustered sampling from the six governorates: Al-Ahmadi (272), Al-Farwaniyah, (246), Hawalli (232), Capital (203), Al-Jahra (144) and Mubarak Al-Kabeer (203). The total numbers of Kuwaitis in each governorate were proportional to population. This involved randomly selecting houses, diwaniyas (local congressional meetings), government enterprises, colleges and shopping centers within each governorate, and from these, individuals were contacted and given an explanation with regard to the purpose of the study. They were free to refuse participation in the survey. Data were collected anonymously via self-administered questionnaire. Those who agreed to take part in the study were given the questionnaires, which were completed anonymously and collected after completion. They were assured for confidentiality and gave written consent to participate in the study. Incentives were not offered for completion of the questionnaire. Subjects who were *<* 20 or *>* 80 years old and expatriates were excluded.

The questionnaire was adapted from previously used and validated study questionnaire to survey NHPs among consumers in Canada [9]. The questionnaire consisted of five sections, and it contains both open-ended and close-ended questions (Additional file 1). The first section included ten items directed to provide information about the characteristics of the respondents. Section two consisted of two questions to determine awareness and explore familiarity of the participants with NHPs. The third section included eleven questions to determine patterns of use of NHPs among respondents. Section four included eight questions to explore the attitudes toward NHPs, the responses were measured using a 5-point Likert scale (strongly disagree, disagree, neither agree nor disagree, agree and strongly agree). The last section included twelve questions to determine the respondents’ need for information about NHPs, which was achieved in three parts. The first part was designed to explore participants’ opinions on six different statements related to the current available information on NHPs. Second part included an open-ended question about respondents’ trust on the source that will provide accurate information on NHPs. The last part was designed to determine participants’ interest in five different types of information regarding NHPs.

The questionnaire was translated into Arabic and subjected to a process of forward and backward translation. The accuracy and meaning of the translated versions both forward and backward were checked, and recommended amendments where necessary were discussed before being finalized. It was pretested for content, design, readability, and comprehension on 10 Kuwaiti subjects, and modifications were made as necessary so that the questionnaire was simple to understand and answer, yet gave accurate data.

Data were entered into the Statistical Package for Social Sciences (SPSS, version 19, SPSS, Chicago, IL, U.S.A.) and descriptive analysis conducted. The results were reported as percentage (95% confidence interval), mean (standard deviation) and median. As the main outcome measure were binary variables describing awareness, familiarity, use and attitudes toward NHPs, logistic regression models were performed using SPSS to fit the best model for the predictor-independent variables. Only the results of multivariate logistic analysis are reported showing odds ratio (OR) and 95% confidence interval. Statistical correlational analysis (Spearman’s r) was used to measure the association between awareness and use of NHPs. Statistical significance was accepted at p *<*0.05. The predictor variables were categorized as follows: (1) gender: males and females; (2) age: young adults [20–39 years], middle age [40–59 years], and old age [*≥* 60 years]; (3) level of education (a) low-intermediate [0–12 years] for those who completed secondary school or less and (b) high [*>*12 years] for those who had diploma, or bachelor degree or postgraduate degree; (4) monthly income: low [< 500 Kuwaiti Dinars (KD)], middle [500–1000 KD] and high [> 1000 KD]; (5) personal health: excellent/very good and good/Fair; and (6) chronic disease: yes had a chronic disease and no chronic disease.

## Results

### Study population

A total of 1300 Kuwaiti subjects were approached to be included in the study, 1173 agreed to participate, giving a response rate of 90.2%. Their mean (SD) age was 40.8 (15.3) years. Four hundred and one (34.2%) individuals reported to have chronic diseases including hypertension (n = 184; 14.7%), diabetes (n = 172; 14.7%), heart disease (n = 71; 6.1%) and other diseases such as asthma, hypothyroidism, chronic kidney disease, epilepsy, parkinsonism and arthritis (n = 197; 16.8%). Table 1 shows the detailed characteristics of the respondents.

**Table 1:** Characteristics of study participants (n = 1173)

**Characteristics**	**Frequency (%)**
**Gender**	
Male	512 (43.6)
Female	661 (56.4)
**Martial state**	
Single	385 (32.8)
Married	754 (64.3)
Divorced	34 (2.9)
**Age (years)**	
20–39	531 (45.2)
40–59	436 (37.2)
≥ 60	206 (17.6)
Educational level*	
Low-intermediate education	159 (13.6)
High education	1010 (86.1)
**Residence (Governorates)**	
Al-Ahmadi	249 (21.2)
Hawalli	204 (17.4)
Al-Farwaniyah	208 (17.2)
Capital	193 (16.5)
Mubarak Al-Kabeer	188 (16.0)
Al-Jahra	131 (11.7)
Monthly income*	
< 500 Kuwaiti Dinars (KD)	258 (22.0)
500–1000 Kuwaiti Dinars (KD)	451 (38.4)
> 1000 Kuwaiti Dinars (KD)	453 (38.6)
**Personal health**	
Excellent	463 (39.5)
Very good	487 (41.5)
Good	192 (16.4)
Fair	31 (2.6)
**Chronic disease**	
Yes	401 (34.2)
No	772 (65.8)

*Percentages may not total 100 because of missing response.

### Awareness and familiarity regarding natural health products

Natural health products (NHPs) were thought to be synonymous with herbal remedies by approximately two-thirds of responders (n = 745; 63.5%), followed by vitamins/minerals (n = 475; 40.5%), traditional medicines (n = 247; 21.1%), probiotics (n = 175; 14.9%), amino acids and essential fatty acids (n = 84; 7.2%), and homeopathic medicines (n = 66; 5.6%). Twenty eight (2.4%) respondents thought that NHPs were other products such as honey, green tea, dairy products and organic food. The belief that NHPs were vitamins/minerals, probiotics, amino acids and essential fatty acids, and homeopathic medicines was found to be significantly less among females compared to males (p < 0.05). Odds ratios (95% CI) were 0.69 (0.53-0.89), 0.70 (0.49-0.90), 0.42 (0.26-0.69) and 0.36 (0.20-0.63), respectively. The acceptance that NHPs were herbal remedies was significantly common among females compared to males (OR: 1.66, 95% CI: 1.28-2.15). The belief that NHPs were amino acids and essential fatty acids was significantly higher among youngest age group (OR: 3.47; 1.44-6.40) and high income earners (OR: 1.70; 1.09-3.15). Higher income and higher level of education were found to be significantly associated with the credence that NHPS were probiotics and homeopathic medicine (p < 0.05).

Most of the respondents were between not at all familiar (n = 226; 19.3%) and somewhat familiar (n = 887; 75.6%) with NHPs, with a mean (SD) of 1.9 (0.5) [where 1 is not at all familiar, 2 is somewhat familiar, and 3 is very familiar]. Familiarity with NHPs was significantly associated with age (p = 0.02), it was significantly higher among middle aged respondents 40–59 years (OR: 1.55; 1.08-2.21) compared to younger and older respondents. There were no significant associations between the other independent variables and familiarity with NHPs (p > 0.05).

### Use of natural health products

Natural health products (NHPs) were used by eight hundred and thirty eight (71.4%; 95% CI: 68.8-74.0) respondents, and was found to be significantly common among females compared with males. There were no significant associations between the use of NHPs and other independent variables (p > 0.05). Table 2 shows the use of NHPs according to differences by the study population characteristics. The frequencies of NHPs use among consumers were as follows: during certain season (n = 397; 47.4%), daily (n = 235; 28.0%), monthly (n = 116; 13.8%) and weekly (n = 90; 10.7%).

**Table 2:** Association between use of natural health products and respondents’ characteristics

**Characteristics**	**Frequency (%)**	**OR (95% CI)**	**p-value**
**Gender**			<0.001
Male	335 (65.4)	Reference	
Female	503 (76.1)	1.90 (1.44-2.51)	
**Age (years)**			0.347
20–39	369 (69.5)	Reference	
40–59	312 (71.6)	1.14 (0.84-1.54)	
≥ 60	157 (76.2)	1.39 (0.88-2.19)	
**Educational level**			0.620
Low-intermediate education	107 (67.3)	Reference	
High education	728 (72.1)	1.10 (0.75-1.62)	
**Monthly income**			0.202
< 500 Kuwaiti Dinars (KD)	176 (68.2)	Reference	
500–1000 Kuwaiti Dinars (KD)	334 (74.1)	1.29 (0.91-1.83)	
> 1000 Kuwaiti Dinars (KD)	329 (72.6)	1.37 (0.96-1.97)	
**Personal health**			0.258
Excellent	320 (69.1)	Reference	
Very good	348 (71.5)	1.11 (0.83-1.49)	
Good/Fair	170 (76.2)	1.41 (0.94-2.13)	
**Chronic disease**			0.798
Yes	297 (74.1)	Reference	
No	541 (70.1)	0.96 (0.69-1.3)	

OR: Odds ratio, CI: confidence interval.

The most commonly used NHPs among respondents were found to be herbal remedies (n = 485; 41.3%) and vitamins/minerals (n = 391; 33.3%). The less commonly used were amino acids and essential fatty acids (n = 133; 11.3%) and probiotics (n = 102; 8.7%). The least used NHPs were traditional medicines (n = 62; 5.3%) and homeopathic medicines (n = 18; 1.5%). Seventeen respondents (1.4%) reported the use of other NHPs such as honey, green tea, dairy products and organic food. The respondents beliefs about NHPs and their use were positively correlated (p < 0.001). The use of vitamins/minerals, probiotics, amino acids and essential fatty acids and homeopathic medicines was found to be significantly higher among males compared to females (p < 0.05), while that of herbal remedies was significantly common among females compared to males (p <0.001) (OR: 2.09; 1.61-2.71). Higher income and high level of education were found to be significantly associated with the use of probiotics and homeopathic medicines (p < 0.05).

The most common reasons for using NHPs were to promote and maintain health (n = 397; 33.8%) and to prevent illness and build immune system (n = 340; 29.0%); while the less common reasons were to increase energy levels (n = 256; 21.8%) and treat specific disease (n = 204; 17.4%). However, the least leading cause for NHPs use was as dietary supplements (n = 133; 11.3%).

The most common source for obtaining NHPs was at the pharmacy (n = 370; 31.5%), followed by health products store (n = 333; 28.4%), supermarket (n = 267; 22.7%) and family members and/or friends (n = 134; 11.4%). The most common sources for recommending and providing information regarding the use of NHPs were family members and/or friends (n = 607; 48.6%) and mass media (n = 345; 27.6%); and the less common sources were from the pharmacists (n = 109; 8.7%), medical doctors (n = 97; 7.8%), dieticians (n = 83; 6.7%) and nurses (n = 6; 0.6%).

One hundred and fifty (17.9%) of NHPs users had experienced a side effect or reaction resulting from the use of NHPs. The most common side effects were nausea (38.0%), diarrhea (24.7%), constipation (20.0%), dizziness (16.0%), vomiting (12.7%), anxiety (9.3%) and skin rash (10.8%). Over two-thirds (n = 102; 68.0%) of them had reported their side effects to family members and/or friends (48.2%), retailers of health products stores (27.9%), pharmacists (18.6%) and medical doctors (5.3%).

Almost three-in-ten (n = 335; 28.6%; 95% CI: 26.0-31.3) participants reported that they had never used NHPs. The most common reasons for not using NHPs were the lack of sufficient information about NHPs (n = 186; 55.5%), their beliefs that they are healthy and no need for NHPs use (n = 100; 29.9%), and the fact that they did not believe on the efficacy of NHPs (n = 49; 14.6%).

### Attitude towards natural health products

Table 3 shows the respondents’ attitudes towards NHPs. Respondents generally agreed on the following statements: i) the Ministry of Health in Kuwait should regulate the claims made by the manufacturers of NHPs (n = 962; 82.0%), ii) it is important to talk to a medical doctor or a pharmacist before using NHPs (n = 890; 75.9%), iii) NHPs can be used to promote and maintain health (n = 846; 72.1%), iv) NHPs are safe because they are made from natural ingredients (n = 720; 61.4%) and v) NHPs can be used to treat illness (n = 702; 59.8%). All values were significantly greater than those who generally disagreed or neutral (p < 0.001). Respondents generally disagreed or were neutral on the following statements: i) NHPs are better for me than conventional medicines (n = 705; 60.1%), ii) a lot of the health claims made by the manufacturers of NHPs are unproven (n = 678; 57.8%) and iii) if a NHP is for sale to the public, I am confident that it is safe (n = 664; 56.6%). All values were significantly greater than those who generally agreed (p < 0.001).

**Table 3:** Respondents’ attitudes towards natural health products (n = 1173)

**Statement**	**Strongly disagree % (95% CI)**	**Disagree % (95% CI)**	**Neutral % (95% CI)**	**Agree % (95% CI)**	**Strongly agree % (95% CI)**	**p value (d + e vs a + b + c)**	**Median**
	**(a)**	**(b)**	**(c)**	**(d)**	**(e)**		
NHPs can be used to help maintain and promote health	4.5 (3.4 - 5.9)	8.2 (6.7 - 9.9)	15.2 (13.2 - 17.4)	52.6 (49.7-55.5)	19.5 (17.3 -21.9)	P < 0.001*	4
NHPs can be used to treat illness	3.6 (2.6 - 4.9)	13.3 (11.4 - 15.4)	23.3 (20.9- 25.8)	49.3 (46.4 - 52.2)	10.6 (8.9 - 12.5)	P < 0.001*	4
I think that NHPs are safe because they are made from natural ingredients	5.3 (4.1 - 6.8)	13.2 (11.4 - 15.3)	20.1 (17.9- 22.6)	43.5 (40.6 - 46.4)	17.9 (15.8 - 20.2)	P < 0.001*	4
If a NHPS is for sale to the public, I am confident that it is safe	7.2 (5.8 - 8.8)	21.5 (19.2 - 24.0)	28.0 (25.4- 30.6)	31.9 (29.2 - 34.7)	11.5 (9.8 - 13.5)	P < 0.001^	3
I think that NHPs are better for me than conventional medicines	8.5 (7.0 - 10.3)	20.5 (18.2 - 22.9)	31.1 (28.5- 33.9)	29.2 (26.6 - 31.9)	10.7 (9.1 - 12.7)	P < 0.001^	3
I think that a lot of the health claims made by the manufacturers of NHPs are unproven	4.3 (3.3 - 5.7)	13.8 (11.9 - 16.0)	39.6 (36.8- 42.5)	32.1 (29.4 - 34.8)	10.1 (8.5 - 12.1)	P < 0.001^	3
The Ministry of Health in Kuwait should regulate the claims made by the manufacturers of NHPS	8.0 (6.6 - 9.8)	3.8 (2.8 - 5.1)	6.2 (4.9- 7.8)	27.0 (24.5 - 29.7)	55 (52.1 - 57.9)	P < 0.001*	5
I think that it is important to talk to a medical doctor or a pharmacist before using NHPs	3.5 (2.6 - 4.8)	8.9 (7.3 - 10.7)	11.8 (10.0-13.8)	36.7 (26.6 -31.9)	39.1 (36.3 - 42.0)	P < 0.001*	4

Measured on a 5-point Likert scale: 1 = Strongly disagree; 2 = disagree; 3 = Neither disagree nor agree (neutral); 4 = Agree; 5 = Strongly agree.

*(d) + (e) significantly greater than (a) + (b) + (c) ^(a) + (b) + (c) significantly greater than (d) + (e).

The percentages of females who generally agreed on the following statements were significantly higher than males (p < 0.05): i) NHPs can be used to promote and maintain health (OR: 1.79; 1.35-2.36), ii) NHPs can be used to treat disease/illness (OR: 1.46; 1.13-1.89), iii) NHPs are safe because they are made from natural ingredients (OR: 1.50; 1.16-1.94), and iv) the Ministry of Health in Kuwait should regulate the claims made by the manufacturers of NHPs (OR: 1.60; 1.16-2.21). The percentages of those with low-intermediate education who generally agreed on the following statements were significantly higher than those with high education (p < 0.05): i) NHPs are safe because they are made from natural ingredients (OR: 1.59; 1.09-2.32), ii) if a NHP is for sale to the public, I am confident that it is safe (OR: 1.83; 1.28-2.61) and iii) NHPs are better for me than conventional medicines (OR: 1.70; 1.19-2.43). The agreement that NHPs can be used to promote and maintain health and NHPs are safe because they are derived from natural ingredients was significantly common among middle and high income groups compared to low income group (p < 0.05). Middle and old age groups significantly generally agreed that NHPs can be used to promote and maintain health and NHPs can be used to treat disease/illness compared with younger group (p < 0.05).

### Information requirements

Table 4 shows the respondents’ opinions regarding the currently available information on NHPs. Respondents generally agreed on the following statements: i) I need more information on NHPs (n = 972; 82.9%) and ii) there is no enough information on NHPs labels to help me understand the products (n = 703; 59.9%). These values were significantly greater than those who generally disagreed or were neutral (p < 0.001). Respondents generally disagreed or were neutral on the following statements: i) the Ministry of Health in Kuwait does a good job of informing the public about NHPs (n = 949; 80.9%), ii) I don’t trust the information on the labels of NHPs (n = 814; 69.4%) and iii) consumers have enough information to make informed decisions about the NHPs that they purchase (n = 792; 67.5%). These values were significantly greater than those who generally agreed (p < 0.001).

**Table 4:** Respondents’ opinions regarding the current available information on natural health products (n = 1173)

**Statement**	**Strongly disagree % (95% CI)**	**Disagree % (95% CI)**	**Neutral % (95% CI)**	**Agree**	**Strongly agree % (95% CI)**	**p value (d + e vs a + b + c)**	**Median**
	**(a)**	**(b)**	**(c)**	**(d)**	**(e)**		
There is no enough information on NHPS labels to help me understand the Products	6.7 (5.3 - 8.3)	14.6 (12.6 - 16.8)	18.8 (16.7 - 21.2)	42.7 (39.9 - 45.6)	17.2 (15.1 - 19.5)	P < 0.001*	4
I don’t trust the information on the labels of NHPs	4.8 (3.7 - 6.2)	28.7 (26.2 - 31.4)	35.9 (33.2 - 38.7)	24.6 (22.1 - 27.1)	6.1 (4.8 - 7.6)	P < 0.001^	3
I need more information on NHPs	4.1 (3.1 - 5.4)	6.3 (5.0 - 7.9)	6.7 (5.3 - 8.3)	50.1 (47.2 - 53.0)	32.8 (30.2 - 35.6)	P < 0.001*	4
Consumers have enough information to make informed decisions about the NHPs that they purchase	10.6 (8.9 - 12.5)	34.5 (31.8 - 37.3)	22.4 (20.1 - 24.9)	26.2 (23.7 - 28.8)	6.3 (5.0 - 7.9)	P < 0.001^	3
Ministry of Health in Kuwait does a good job of informing the public about NHPs	24.6 (22.1 - 27.1)	31.2 (28.6 - 34.0)	25.2 (22.7 - 27.8)	12.6 (10.8 - 14.7)	6.4 (5.2 - 8.1)	P < 0.001^	2

Measured on a 5-point Likert scale: 1 = Strongly disagree; 2 = disagree; 3 = Neither disagree nor agree (neutral); 4 = Agree; 5 = Strongly agree.

*(d) + (e) significantly greater than (a) + (b) + (c).

^(a) + (b) + (c) significantly greater than (d) + (e).

The most commonly trusted sources to provide information on NHPs was the pharmacist (n = 663; 56.5%), followed by the medical doctor (n = 485; 41.3%) and the dietician (n = 390; 33.2%). Less commonly trusted sources were family member and/or friend (n = 209; 17.8%), the retailer of health product store (n = 92; 7.8%), coaches in the club (n = 58; 4.9%) and NHPs manufacturers (n = 55; 4.7%). Twenty six (2.2%) respondents indicated the nurse as the least commonly trusted person to provide information on NHPs.

Respondents have shown high interest in obtaining different types of information related to NHPs. Almost nine-in-ten respondents indicated their interest in getting information on the following: i) how to safely use NHPs (n = 1112; 94.8%), ii) uses and beneficial effects of NHPs (n = 1109; 94.5%); iii) potential side effects of NHPs (n = 1072; 91.2%), iv) how to report unwanted side effects (n = 1064; 90.7%), and v) possible drug interactions (n = 1062; 90.5%).

## Discussion

This is the first known study to demonstrate the levels of awareness, patterns of use and attitudes toward NHPs in the Kuwaiti population. The current results would be the first step in providing a quantitative measurement of the prevalence, socio-cultural and personal factors underlying an individual’s decision to use a NHP in Kuwait, and could be utilized in designing educational campaigns to promote the safe use of NHPs and to minimize risks associated with their usage among the general population.

The present findings indicate that a high percentage of Kuwaitis surveyed are unclear about what are NHPs. About two-thirds of respondents accurately identified herbal remedies as NHPs, however, a high proportion of Kuwaitis are less certain regarding vitamins or minerals, traditional medicines, probiotics, amino acids and essential fatty acids and homeopathic medicines as NHPs. Only 5.1% reported that they are very familiar with NHPs. These findings are in contrast with that reported in North America where about three-quarters of the respondents are knowledgeable about vitamins/minerals, herbal remedies, essential fatty acids and homeopathic medicines as NHPs; and 23% were very familiar with NHPs [16]. This demonstrates inadequacy in formal education about NHPs in Kuwait, which could be due to the lack of and/or insufficient dissemination of informed knowledge by health officials to the general public. This is confirmed by the findings that 83% of respondents indicated their desire for more information on NHPs; and that the major sources for providing information regarding NHPs use were family members and/or friends and the mass media. The higher awareness claimed by the respondents regarding herbal remedies might be due to the cultural background in the Eastern Mediterranean Region, which encourages the use of these remedies [5,6,8-14,23-25,41].

In the current study, the percentage of having ever used a NHP is high (71.4%), which is close to that reported in Canada [15,16], but higher than that for the general populations in the USA, South Australia and Wales [17,18,20,21]. In this study, it was not surprising that the most commonly used NHP is herbal remedies, since the use of herbal products is very common in the Eastern Mediterranean Region [5,6,8-14,23-25,41]. This may also be attributed to religious and cultural beliefs and family factors in the Arab world that are different from those prevalent in the west, where vitamins and minerals continue to be the most widely used NHPs [15-17,19]. The present finding that females are more likely to use NHPs is in accordance with other studies [5,9,15,16,19,20,41]. It has been reported that females have more positive attitudes and enthusiasm with regard to CAM, which might explain this higher use of CAM [43]. About three-quarters of respondents with chronic diseases indicated their use of NHPs, and about half of them used herbal remedies. This finding is close to the rates observed in other studies in the Eastern Mediterranean Region [5,6,9,12]. However, it is not known in this study whether these remedies were being used as an adjunct to prescribed medicines or whether they were the sole means of treatment. If the latter, this gives rise for concern in view of the morbidity potential arising from inadequate disease control. If the former, this is an area of great concern due to the possibility for adverse interactions [23,35,36].

The present findings indicate that the primary reasons for using NHPs were to maintain and promote health and to prevent illness and build the immune system, which are in agreement with that reported from Canada and South Australia [16,20]. This could be explained by the respondents agreement that NHPs could be used to promote and maintain health (72.1%) and NHPs are safe because they are made from natural ingredients (61.4%). The widespread perception among the users that the NHPs are safe and not to be associated with risk was reported by other studies [30,31]. Although NHPs are routinely available without prescription that does not indicate they are completely safe for all individuals, especially when used without professional guidance. Adverse drug reactions have been reported when herbal medicines are used alone or concurrently with conventional medicines [32,33,35,36].

In this study, while 76% of the respondents indicated that it is important to talk to a medical doctor or pharmacist before using NHPs, about half of the users self-select NHPs based on potentially doubtful or unproven information that they receive from family members and/or friends, or the mass media. These findings are comparable to those reported by previous studies [5,6,9,19,26]. The current results indicate that the role of healthcare practitioners (medical doctor and pharmacist) is not currently evident. Faced with the increasing demand for CAM including NHPs by the patients, it is crucial that healthcare providers be prepared to discuss their uses and limitations, as well as possible side effects or adverse reactions [28,34]. The introduction of CAM education in the undergraduate curriculum of Medical and pharmacy schools in Kuwait will equip students with knowledge on CAM enabling them to advise patients during professional practice [41]. The present results show that the main source for obtaining NHPs was community pharmacy and the most trusted person to provide information about NHPs is the pharmacist. A recent study conducted in the US reported that pharmacists are now receiving more questions from patients regarding the use of NHPs than ever before, which necessitate that pharmacists become more knowledgeable about these products and their uses, dosing, adverse effects, drug interactions and contraindications [44].

Few (17.9%) of those who use NHPs indicate that they have experienced certain associated side effects, which is higher than that reported in North America [15,16,45]. Most of them reported these side effects to family members and/or friends and retailers from health product stores, which is in accordance with that reported in North America [46]. The high interest indicated by the respondents to acquire information with respect to the potential side effects and how to report unwanted side effects highlighted the need for the establishment of an active reporting system in Kuwait. A national reporting system is important to encourage healthcare practitioners, health products stores personnel and consumers to assume responsibility for reporting side effects or reactions in order to improve the pharmacovigilance of NHPs. Most of the participants agreed that it is important to talk to a medical doctor or a pharmacist before using a NHP, and showed high interest in obtaining information on the uses and beneficial effects of NHPs and how to be safely used. It was reported that the consumers’ comfort level with their healthcare providers influences their inclination to report use and experience of side effects or adverse reactions associated with NHPs [46]. Thus, further research is needed to understand how healthcare providers and users in Kuwait discuss NHPs, what benefits and adverse reactions consumers experience, and how best to be advised.

The present findings show a notably high level of agreement among the respondents that the Ministry of Health in Kuwait should consider to be more involved in regulating the claims made by the manufacturers of NHPs. The advertisement of false claims could cause consumers to modify or discontinue the use of their prescribed drugs without appropriate medical advice. This may jeopardizes the users’ safety and places an urgent mandate on health authorities to put a proper regulatory plan in place. It was reported that without appropriate regulation, NHPs users could be exposed to several degrees of risks ranging from inappropriate usage to severe life-threatening adverse reactions [34]. Although regulations exist in Kuwait since 1989, but its enforcement is pivotal to protect users against false and misleading claims; possible manipulation of safety test results; and interactions between NHPs or between NHPs and medications [47]. A collaboration between healthcare practitioners, nutritionists and regulatory toxicologists in Kuwait to plan and implement proper regulations will offer the ultimate health and therapeutic benefits to the NHPs consumers. As the popularity of CAM is growing among the general populations, there is a need for its closer integration into the health care system in Kuwait [28,47]. The Gulf countries acknowledged the importance of setting a unified Gulf strategy and appropriate policies and procedures to organize, regulate and monitor the practice of CAM [47].

High willingness shown by respondents to learn more about different types of NHPs. Furthermore, they declared that the Ministry of Health in Kuwait needs to be more proactive in informing the public about NHPs and that there is not enough information on NHPs labels to help them understand the products. These would certainly require an active role for the Ministry of Health to serve the best interest of the public through focusing on the dissemination of unbiased information about the risks and benefits of NHPs [47]. Provision of detailed information on product labels, including the names and amounts of all ingredients, the medicinal claim, instructions for use, cautions, adverse effects and potential drug interactions will help consumers to make more informed decisions about their health prior to the purchase a NHP. The healthcare providers could use this information to advise patients more effectively about the safe use of NHPs.

### Strengths and limitations

The strength of this study is that we used appropriate sampling method and sample size to generate a representative data about the study population. Further strength is the high response rate. The results can, therefore, be generalized at the study population level in Kuwait. We acknowledge that this type of study, using a self-administered questionnaire, has its limitations. It depends very much upon information given by respondents and open to recall bias or error. The extent of truthful answers or verifying respondents’ claims is not possible in this type of study, which were taken at face value. A further limitation of the study is the cross-sectional nature of the data that represented one point in time and, therefore, do not reflect any changes in respondents’ beliefs over time in relation to NHPs.

## Conclusions

This is the first study to demonstrate the levels of awareness, patterns of use and attitudes toward NHPs in Kuwait. Its findings allow for important comparative work with existing and future investigations in middle-eastern countries. Given the growing global use of NHPs and documented health issues related to inappropriate practices of such products, the study findings will help policy makers, health services to understand NHPs consumption across Kuwait and to plan for appropriate policies and procedures to organize, regulate and monitor the practice of CAM therapies including NHPs.

## Abbreviations

NHPs: Natural health products; CAM: Complementary and alternative medicine; TM: Traditional medicine; WHO: World Health Organization; CI: Confidence interval; SD: Standard deviation.

## Competing interests

The author declares that they have no competing interests.

## Authors’ contributions

AA designed and supervised the study, performed the data analysis and wrote the manuscript. DA contributed in development and pre-testing of the study questionnaire, data collection and reviewed the manuscript. Both authors read and approve the final manuscript.

## Pre-publication history

The pre-publication history for this paper can be accessed here:

http://www.biomedcentral.com/1472-6882/14/105/prepub

## Supplementary Material

Additional file 1 Questionnaire to investigate the natural health products use among the public in Kuwait. Click here for file
