# Supplementary material for: Public awareness, patterns of use and attitudes toward natural health products in Kuwait: a cross-sectional survey
Source: BMC Complement Altern Med. 2014 Mar 19;14:105. doi: 10.1186/1472-6882-14-105 (PMC3999934; doi:10.1186/1472-6882-14-105)
Supplement: Additional file 1 — Questionnaire to investigate the natural health products use among the public in Kuwait. [file 1472-6882-14-105-S1.docx]

**Questionnaire to investigate the natural health products use among the public in Kuwait**

**A. Demographic and other Characteristics**

**PLEASE FILL IN OR TICK (√) THE APPROPRIATE ANSWER**

1. Gender: □ Male □ Female

2. Age (in years): _______

3. Marital status: □ Single □ Married □ Divorced

4. Educational level: □ Uneducated □ Primary school □ Intermediate school □ Secondary school

□ Diploma □ University □ Postgraduate studies

5. Employment: □ Unemployed □ Labor □ Professional □ Business □ Clerical □ Student

□ Housewife □ Retired

6. Residence: □ Capital □ Hawalli □ Farwanhiya □ Ahmadi □ Al-Jahra

□ Mubarak Al-Kabeer

7. Monthly Income: □ Less than 500 KD □ 500-1000 KD □ Greater than 1000 KD

8. Personal Health: □ Excellent □ Very good □ Good □ Fair □ Poor

9. Do you suffer any chronic disease? □ Yes □ No

10. If yes to Q9, which of the following chronic diseases do you have?

□ Hypertension □ Diabetes □ Heart diseases □ Others (Specify)

**B. Knowledge about natural health products:**

11. What do you think natural health products are? **Please tick (√) all that apply**

□ Vitamins/Minerals □ Herbal remedies □ Homeopathic medicines □ Traditional medicines

□ Probiotics □ Aminoacids and essential fatty acids □ Others(specify) ........................

12. Please indicate how familiar are you with natural health products? **Please** **tick** **(√) only** **one** **option**

□ Not at all familiar □ Somewhat unfamiliar □ Very familiar

**C. Pattern of use of natural health products**:

13. Have you ever used a natural health product? □ Yes □ No

**If NO to Question 13 skip to Question 23**

14. If yes to Q13, which one of the following best describe your use of a natural health product? **Please** **tick** **(√) only** **one** **option**

□ Daily □ Weekly □ Monthly □ Only during certain season

15. Which natural health product have you used? **Please tick (√) all that apply**

□ Vitamins/Minerals □ Herbal remedies □ Homeopathic medicines □ Traditional medicines

□ Probiotics □ Aminoacids and essential fatty acids □ Others (specify)…….

16. Why did you decide to use a natural health product? Please tick all that apply

□ To help promote and maintain health □ To prevent illness/ build immune system

□ To treat specific disease/ symptom □ To supplement what I'm lacking in my diet

□ To increase my energy levels □ Others ( specify)…………………

17. What is the source from which you obtained the natural health product? **Please tick (√) all that apply**

□ Pharmacy □ Health products store □ Supermarket □ Family member/Friend

□ Others (Specify)……………

18. Who recommended and provided you with the information regarding the use of the natural health product you had taken? **Please tick (√) all that apply**

□ Family member/Friend □ Medical Doctor □ Pharmacist □ Nurse □ Dietician

□ Mass Media (TV/Radio/Newspaper □ Others (specify)………………….

19. Have you ever experienced an unwanted side effect or reaction when using a natural health product?

□ Yes □ No

**If NO to Question 19 skip to Question 24**

20. What type of unwanted side effect or reaction did you experience? **Please tick (√) all that apply**

□ Nausea □ Vomiting □ Diarrhoea □ Constipation □ Nervousness/Anxiety □ Dizziness

□ Skin rash □ Others (Specify) ………………………

21. Did you report this unwanted side effect or reaction of the natural health product to any one?

□ Yes □ No

**If NO to Question 21 skip to Question 24**

22. Who did you report unwanted side effect or reaction to? **Please tick (√) all that apply**

□ Medical doctor □ Pharmacist □ Family member/Friend □ Health product personnel

□ Others (Specify)………………………………

23. If you have never used a natural health product, what is the main reason? **Please** **tick** **(√) only** **one** **option**

□ I do not believe in the efficacy of the natural health product

□ I am healthy and no need for its use

□ I don't know enough information available about natural health products

□ Natural health products are expensive for me to use

□ Others(specify)………………………………

**D. Attitudes towards natural health products:**

**Please insert tick (√) in the corresponding box that most appropriately reflects your response**

| **Statement** | **1.Strongly Disagree** | **2. Disagree** | **3. Neither Agree nor Disagree** | **4. Agree** | **5.Strongly Agree** |
| --- | --- | --- | --- | --- | --- |
| 24. NHPs can be used to help maintain and promote health |  |  |  |  |  |
| 25. NHPs can be used to treat illness |  |  |  |  |  |
| 26. I think that NHPs are safe because they are made from natural ingredients |  |  |  |  |  |
| 27. If a NHP is for sale to the public, I am confident that it is safe |  |  |  |  |  |
| 28. I think that NHPs are better for me than conventional medicines |  |  |  |  |  |
| 29. I think that a lot of the health claims made by the manufacturers of NHPs are unproven |  |  |  |  |  |
| 30. The Ministry of Health in Kuwait should regulate the claims made by the manufacturers of NHP |  |  |  |  |  |
| 31. I think that it is important to talk to a medical doctor or pharmacist before using NHPs |  |  |  |  |  |

**E: Information Requirements**

**Please insert tick(/) in the corresponding box that most appropriately reflects your opinion**

| **Statement** | **1. Strongly Disagree** | **2. Disagree** | **3. Neither Agree nor Disagree** | **4. Agree** | **5.Strongly Agree** |
| --- | --- | --- | --- | --- | --- |
| 32. There isn't enough information on NHP labels to help me understand the  Products |  |  |  |  |  |
| 33. I don't trust the information on the labels of NHPs |  |  |  |  |  |
| 34. I need more information on NHPs |  |  |  |  |  |
| 35. Consumers have enough information to make informed decisions about the NHPs that they buy |  |  |  |  |  |
| 36. Ministry of Health in Kuwait does a good job of informing the public about NHPs |  |  |  |  |  |

37. Who do you trust to provide accurate information on natural health products? **Please tick (√) all that apply**

□ Medical Doctor □ Pharmacist □ Nurse □ Dietician □ Natural health product manufacturer □ Family member/Friend □ Health products store personnel □ others (specify)………..

Please indicate how interested you are with each of the following types of information on natural health products: **Please insert tick (√) in the corresponding box that most appropriately reflects your interest**

| **Types of information** | **Not interested** | **Somewhat interested** | **Very interested** |
| --- | --- | --- | --- |
| 38. Uses and beneficial effects of NHPs |  |  |  |
| 39. Potential side effects of NHPs |  |  |  |
| 40. Possible drug interactions |  |  |  |
| 41. How to safely use NHPs |  |  |  |
| 42. How to report unwanted side effect or reaction |  |  |  |
